# Predicting long-term outcomes of kidney transplantation in the era of artificial intelligence

**DOI:** 10.1038/s41598-023-48645-w

**Published:** 2023-12-02

**Authors:** Samarra Badrouchi, Mohamed Mongi Bacha, Abdulaziz Ahmed, Taieb Ben Abdallah, Ezzedine Abderrahim

**Affiliations:** 1 Department of Internal Medicine A, Charles Nicolle Hospital, Tunis, Tunisia; 2 Faculty of Medicine of Tunis, University of Tunis El Manar, Tunis, Tunisia; 3 Laboratory of Kidney Transplantation Immunology and Immunopathology (LR03SP01), Charles Nicolle Hospital, Tunis, Tunisia; 4 Department of Health Services Administration, School of Health Professions, The University of Alabama at Birmingham, Birmingham, AL USA

**Keywords:** Nephrology, Urology

## Abstract

The ability to accurately predict long-term kidney transplant survival can assist nephrologists in making therapeutic decisions. However, predicting kidney transplantation (KT) outcomes is challenging due to the complexity of the factors involved. Artificial intelligence (AI) has become an increasingly important tool in the prediction of medical outcomes. Our goal was to utilize both conventional and AI-based methods to predict long-term kidney transplant survival. Our study included 407 KTs divided into two groups (group A: with a graft lifespan greater than 5 years and group B: with poor graft survival). We first performed a traditional statistical analysis and then developed predictive models using machine learning (ML) techniques. Donors in group A were significantly younger. The use of Mycophenolate Mofetil (MMF) was the only immunosuppressive drug that was significantly associated with improved graft survival. The average estimated glomerular filtration rate (eGFR) in the 3rd month post-KT was significantly higher in group A. The number of hospital readmissions during the 1st year post-KT was a predictor of graft survival. In terms of early post-transplant complications, delayed graft function (DGF), acute kidney injury (AKI), and acute rejection (AR) were significantly associated with poor graft survival. Among the 35 AI models developed, the best model had an AUC of 89.7% (Se: 91.9%; Sp: 87.5%). It was based on ten variables selected by an ML algorithm, with the most important being hypertension and a history of red-blood-cell transfusion. The use of AI provided us with a robust model enabling fast and precise prediction of 5-year graft survival using early and easily collectible variables. Our model can be used as a decision-support tool to early detect graft status.

## Introduction

Chronic Kidney disease (CKD) is a significant global health issue due to its high prevalence and associated risk of progression to end-stage renal disease (ESRD). ESRD affects more than 7 million people worldwide^1^. Kidney transplantation (KT) is the most desired and cost-effective treatment for ESRD, which improves the life quality and survival rates of patients^2^. Despite the increasing number of KTs performed each year, the number of people on waiting lists continues to grow. In 2019, there were more than 113,000 people on the US national kidney transplant waiting list, with an average of one person added every 10 min. Approximately 20 people die every day while waiting for a kidney transplant^3^. Thus, kidney allograft failure is a fatal outcome that contributes to the backflow of people to already-overburdened lists. Improving KT outcomes is therefore crucial.

Medical advancements in surgical techniques and immunosuppressive drugs have improved short-term outcomes of kidney allografts since the early 1980s. However, there has been no significant improvement in long-term graft survival since the 2000s^4–6^. Consequently, there is now a shift in focus toward forecasting the long-term survival of kidney allografts^7^.

An accurate prediction of long-term graft survival can aid nephrologists in understanding the progression of graft function in each patient and providing more personalized monitoring and clinical care. Enhanced prediction of KT outcomes would not only help in daily clinical care, therapeutic decisions making, and counseling of patients but also facilitate conducting clinical trials aiming to assess long-term outcomes^8^. Such studies are needed to assess immunosuppressive drugs that are intended to improve long-term graft survival as the paucity of KT long-term outcomes is partly linked to these drugs. Regulatory agencies and medical societies have highlighted the need for an early reliable alternate tool in transplantation that pertinently predicts long-term graft survival^9^. Prediction of kidney graft survival is difficult due to the diversity and complexity of factors leading to graft failure.

Artificial intelligence (AI), specifically machine learning (ML), is playing an increasingly important role in prediction tasks in medicine by enabling the analysis of complex and big data. ML offers algorithms that can improve prediction accuracy compared to conventional statistical models by capturing complex relationships among variables. They are also efficient in handling data with a large number of variables. Few studies have used advanced ML techniques to build models that predict long-term kidney graft survival. This research aims to investigate the viability of AI techniques to predict long-term kidney graft survival by:

- Using ML algorithms to develop a predictive model for early prediction of long-term kidney graft survival.
- Evaluating the ML-based model using several performance measures.
- Performing parallel classical statistical analysis.

## Methods

### Study design

We conducted a longitudinal research study, using the Charles Nicolle Hospital KT database which contains data collected over 33 years (1986–2019). We included 407 KTs and used a threshold of 5 years to define the long-term survival of kidney transplants. After a preliminary traditional statistical analysis, we developed an ML-based predictive model.

### Definitions

- *Artificial intelligence* AI is a branch of computer science that involves the use of computers to model intelligent behavior with minimal human intervention. AI is widely used in medicine to analyze complex medical data in the diagnosis, treatment, and prediction of outcomes.
- *Machine learning* A subset of AI that focuses on the development of computer programs able to learn from data without explicit programming for a specific task. ML algorithms can learn from data and improve through experience without human intervention.
- *Data preparation* The manipulations and transformations applied to a dataset to make it suitable for analysis by ML algorithms during the training and testing process.
- *Feature selection* The selection of a subset of input variables that are significant in affecting the output of interest (long-term survival). It involves removing irrelevant variables without the loss of predictive information to improve the performance of the model. Various methods can be used, from traditional statistical methods to ML-based methods.
- *Model training* The process of automatically building a model is referred to as “training”. The process of training an ML model involves providing the learning algorithm with training data to learn from. In a supervised learning problem, the training data must contain the correct output that we wish to predict. The learning algorithm learns from the training data the function that matches the input variables to the desired output, resulting in an ML model that captures these patterns. The trained model will be applied to new data for forecasting the likelihood of a particular outcome.
- *Model testing* The process of evaluating a model using data, other than the training data, with a known output and comparing the predictions of the model to the actual outcomes to calculate performance measures.

### Patients and methods

Of all the kidney transplants in the database (1986–2019), we only included those performed before December 2014 to have a minimum follow-up period of 5 years for all KTs. We did not include pediatric transplants, where the recipient was under 15 years old at the time of the transplant. We also excluded transplants with graft failure and return to dialysis within the first month, as well as patients with one or more missing values in any of the variables retained after data preparation. During data analysis, our patients were divided into two groups:

- *Group A* including patients with a functional graft for 5 years or more.
- *Group B* including patients who returned to dialysis in less than 5 years.

### Classical statistical analysis

We conducted a preliminary statistical analysis to gain insight into our database. We described continuous variables by using means and standard deviations. We compared means between groups by using the student’s t-test and we used the Chi-square (chi2) test (or Fisher’s exact test if appropriate) to compare proportions to test whether the difference between the two groups is significant. The null hypothesis was rejected if the p-value was below 5%.

### Machine learning modeling

**Figure 1:**
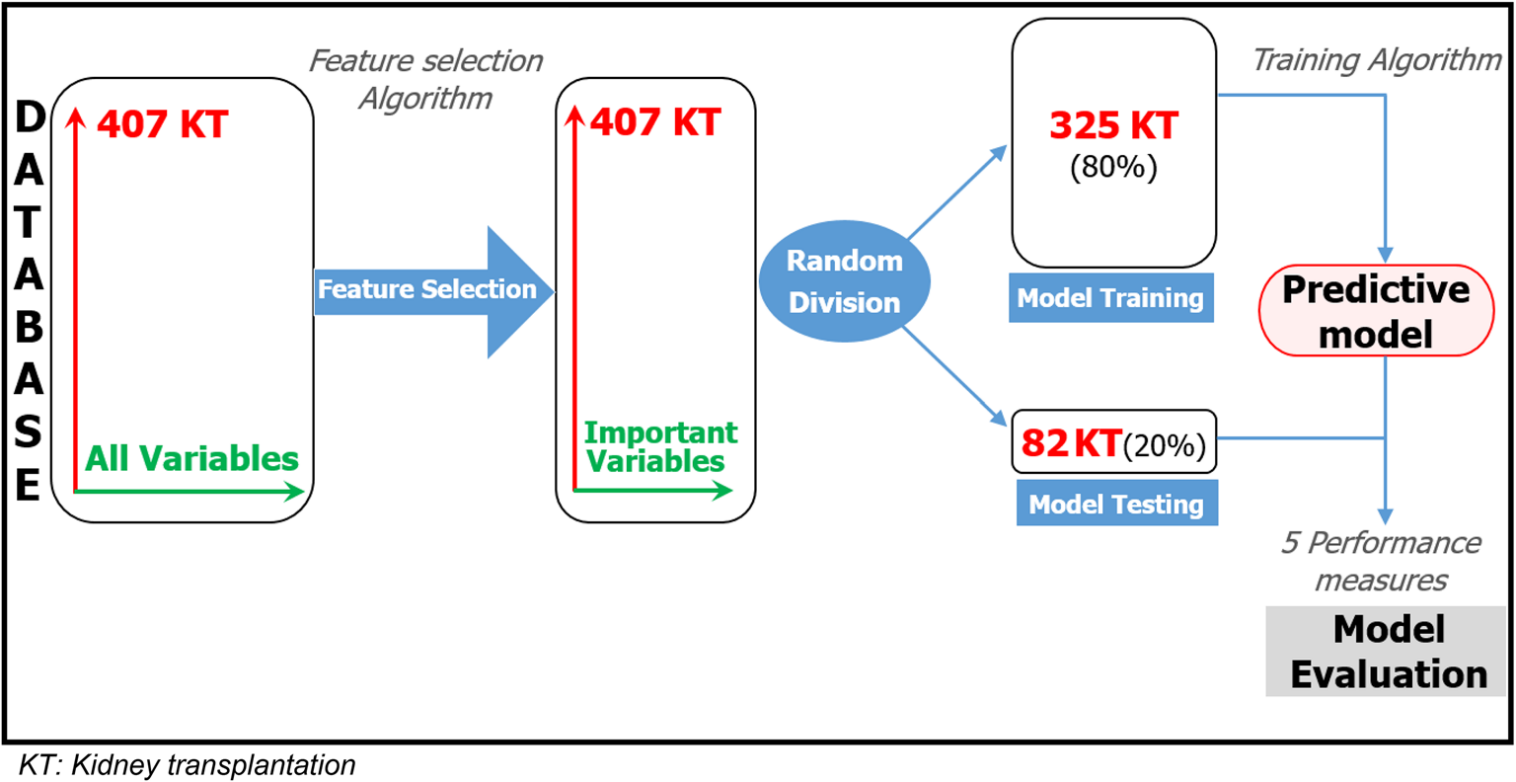
Process followed to develop a predictive model.

We developed a model that enables us to predict whether the graft will survive for at least 5 years or not based on a set of variables (features) selected by an ML algorithm. The robustness of the predictive model was evaluated using several performance measures. The model development consisted of two steps: (1) feature selection as defined previously then (2) the model training on data containing only the selected features. The process followed is summarized in Fig. 1.

#### Feature selection

We tested seven methods. We implemented these methods with ML algorithms: Least Absolute Shrinkage and Selection Operator Logistic Regression (LASSO), Random Forests (RF), Decision Trees (DT), and Chi2.

#### Model training

We tried five training algorithms which are: (1) Artificial Neural Network (ANN), (2) Extreme Gradient Boosting (XGB), (3) K-Nearest Neighbor (KNN), (4) DT, and (5) Logistic regression (LR).

Finally, we trained and tested 35 models. The model with the best performance measures was considered the final one.

#### Model evaluation

After implementing each model, we used the testing set of data to find out how effective the predictions of the model are, based on the correctness of the model’s predictions, we calculated five performance measures which are: (1) sensitivity (Se), (2) specificity (Sp), (3) F1 measure, (4) accuracy, and (5) Area Under Curve (AUC).

### Previous research

The current study builds upon prior research by our team^10^. In that work, we laid the foundation for the ML techniques employed. The previous publication delved deeply into the technical aspect and model development; it was tailored for readers in the ML community. In contrast, the present manuscript has been designed address the medical community, with a specific emphasis on the final retained model’s utility in a clinical setting.

### Ethical considerations

Informed consent from individual patients was waived by the ethics committee of Charles Nicolle Hospital in Tunis (Tunisia) since the analyses were performed on anonymized data from Charles Nicolle Hospital KT database. The anonymity of the patients was maintained throughout the study, and all data were analyzed in a manner that protected patient privacy.

### Ethical approval

This research was carried out following the institutional and national ethical guidelines for human studies and according to the ethical principles outlined in the Declaration of Helsinki. All the procedures in the present study were approved by the ethics committee of Charles Nicolle Hospital in Tunis (Tunisia).

### Ethical statement

The authors of this manuscript certify that this material is their original work and it has not been previously published or submitted for publication elsewhere. All authors have actively contributed to this research and take responsibility for its content.

## Results

### Graft survival

**Figure 2:**
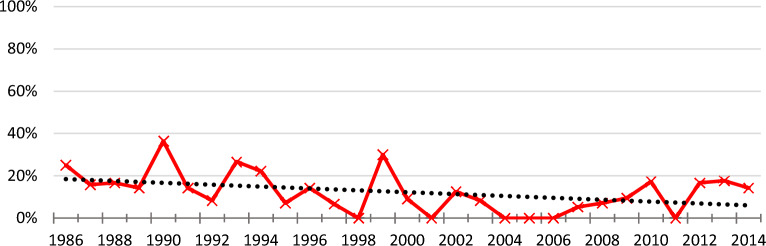
Evolution of the rate of transplantations with poor long-term survival.

Figure 2 shows the evolution of the percentage of KTs with poor graft survival (group B) per year. A maximum of 36% was observed in 1991. The trend line (the black dotted line) shows a decrease in the rate of KTs with poor long-term survival during the study period.

**Figure 3:**
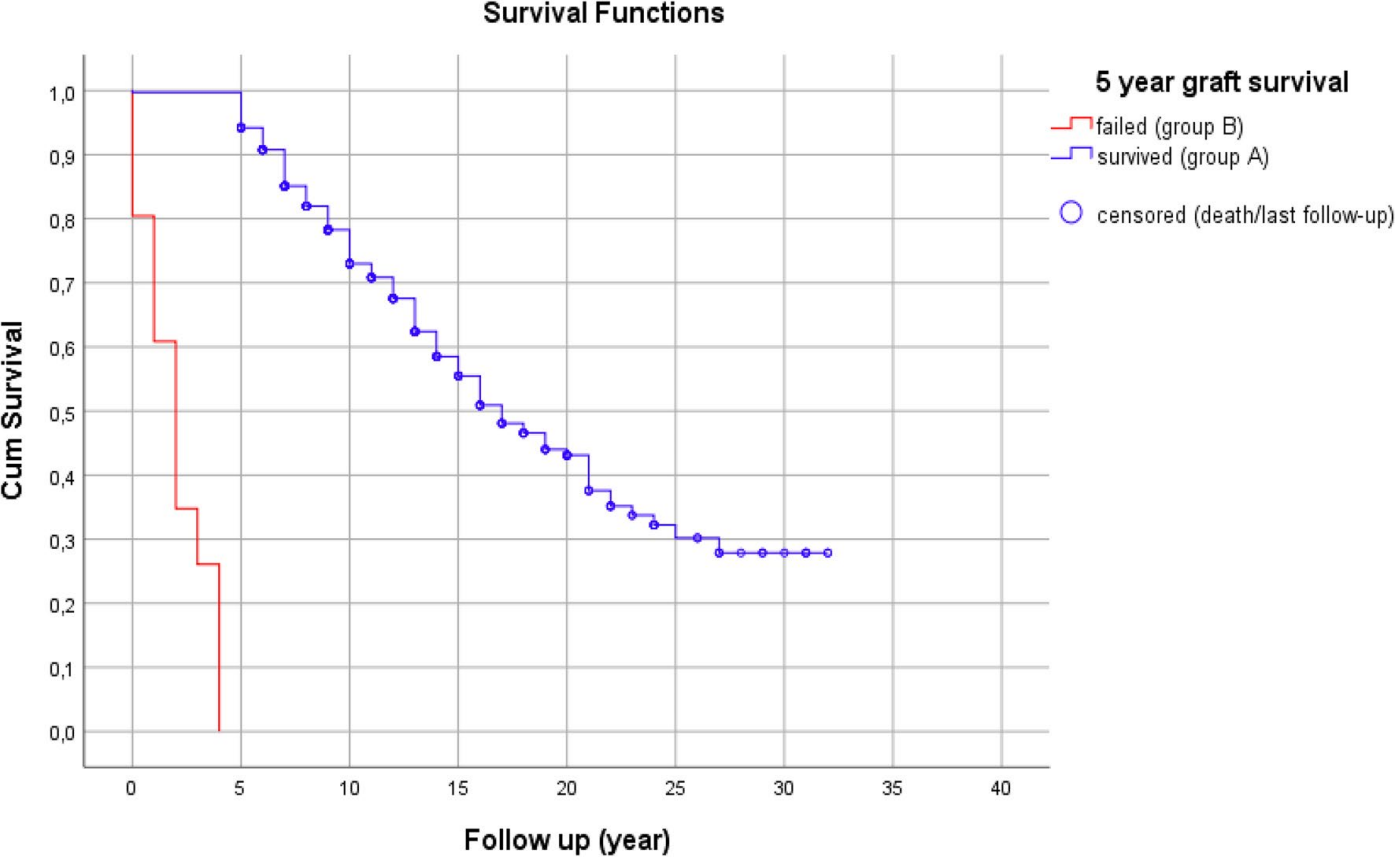
Kaplan–Meier plots of cumulative graft survival of the study groups.

In the overall population, the median survival time was 15 years. The estimated 5-year and 10-year overall cumulative survival rates were 86% and 69%, respectively. In Fig. 3, the red curve shows the cumulative graft survival rate in group B. The median survival time in this group was 2 years. Graft failure occurred during the first 3 years in 70% of them. In group A (the blue curve), more than half of the patients had a functional kidney graft 15 years after KT.

### Classical statistical modeling

Table 1 presents the relative risk of graft failure within 5 years and the corresponding 95% confidence interval (CI) of each one of the considered variables in the univariate analysis.

**Table 1:** Relative risk of 5-year graft failure in the univariate analysis.

	Variables		Group A	Group B	RR	95% CI	p
Recipient	Age/year (mean)		33.4	32	0.986	0.955–1.017	NS
	Gender (%)	Male	66.2	67.4	1	–	–
		Female	33.8	32.6	0.948	0.493–1.823	NS
	Hypertension (%)	No	39.9	32.6	1	–	–
		Yes	60.1	67.4	1.371	0.715–2.631	NS
	Diabetes (%)	No	94.7	97.8	1	–	–
		Yes	5.3	2.2	0.275	0.036–2.072	NS
	Viral hepatits B (%)	Negative	97	93.5	1	–	–
		Positive	3	6.5	2.220	0.596–8.271	NS
	Viral hepatits C (%)	Negative	85.2	86.5	1	–	–
		Positive	14.8	13.5	0.899	0.331–2.443	NS
	Nephropathy (%)	Vascular	8	6	1	–	–
		Glomerular	41.3	43.5	0.649	0.240–1.755	NS
		Tubulo-interstitial	21.6	15.2	0.434	0.135–1.399	NS
		Hereditary	5.8	2.2	0.230	0.026–2.057	NS
		Undetermined	23.3	26.1	0.690	0.238–2.007	NS
	Dialysis modality (%)	PD	19.4	10.9	1	–	–
		HD	74.5	87	2.082	0.792–5.471	NS
	Dialysis duration/year (Mean)		3.5	3.7	1.001	0.994–1.009	NS
	Transfusion (%)	No	38.2	26.1	1	–	–
		Yes	61.8	73.9	1.753	0.878–3.501	NS
	Cytotoxic antibodies (%)	Negative	85.9	82.6	1	–	–
		Positive	14.1	17.4	1.280	0.565–2.899	NS
	Total HLA MM (%)	0	17.5	19.6	1	–	–
		1–2	33.5	32.6	0.868	0.360–2.094	NS
		≥ 3	49	47.8	0.870	0.380–1.990	NS
	HLA- MM A (%)	0 MM	34.9	32.5	1	–	–
		1 MM	56.2	47.8	1.111	0.564–2.190	NS
		2 MM	14.4	15.2	1.096	0.401–2.994	NS
	HLA-MM B (%)	0 MM	29.4	36.9	1	–	–
		1 MM	56.2	47.8	0.676	0.344–1.327	NS
		2 MM	14.4	15.2	0.839	0.328–2.150	NS
	HLA-MM DR (%)	0 MM	40.4	43.5	1	–	–
		1 MM	50.7	47.8	0.878	0.461–1.670	NS
		2 MM	8.9	8.7	0.913	0.292–2.852	NS
Donor	Age/year (mean)		40.1	43.5	1.022	0.997–1.048	NS
	Age (%)	< 45 years	63.2	43.5	1	–	–
		≥ 45 years	36.8	56.5	2.229	1.198–4.147	< 0.02
	Gender (%)	Male	52.9	50	1	–	–
		Female	47.1	50	0.890	0.482–1.644	NS
	Gender match (%) Donor → recipient	M → M	30.5	28.3	1	–	–
		F → F	16.9	10.9	0.694	0.236–2.038	NS
		F → M	35.5	36.9	1.124	0.523–2.417	NS
		M → F	17.1	23.9	1.501	0.635–3.552	NS
	Donor type (%)	Living	84.8	82.6	1	–	–
		Deceased	15.2	17.4	1.171	0.519–2.645	NS
Procedure	Cold ischemia/hour (mean)		21.8	21.4	0.981	0.856–1.124	NS
	Cold ischemia (%)	< 20 h	36.4	25	1	–	–
		≥ 20 h	63.6	75	0.880	0.189–4.085	NS
	Warm ischemia/min (mean)		38.2	40.2	1.018	0.990–1.046	NS
	Warm ischemia (%)	< 30 min	18.6	17.4	1	–	–
		≥ 30 min	81.4	82.6	1.082	0.483–2.427	NS
Immunosuppressive treatment	Induction (%)	Yes	88.9	91.3	1	–	–
		No	11.1	8.7	0.993	0.870–2.662	NS
	Polyclonal anti-lymphocyte (%)	No	20.5	19.6	1	–	–
		Yes	79.5	80.4	1.060	0.490–2.294	NS
	Anti-CD3 (%)	No	99.2	93.5	1	–	–
		Yes	0.8	6.5	8.326	1.629–42.547	< 0.02
	Anti-CD25 (%)	No	91.4	95.7	1	–	–
		Yes	8.6	4.3	0.467	0.108–2.018	NS
	Cyclosporine A (%)	No	43.8	54.3	1	–	–
		Yes	56.2	45.7	0.654	0.353–1.211	NS
	Tacrolimus (%)	No	65.7	60.9	1	–	–
		Yes	34.3	39.1	1.229	0.654–2.309	NS
	Azathioprine (%)	No	63.4	47.8	1	–	–
		Yes	36.6	52.2	1.893	1.021–3.507	< 0.05
	MMF (%)	No	20.8	47.8	1	–	–
		Yes	79.2	52.2	0.286	0.152–0.538	< 0.001
Post-KT	Length of 1st hospitalization/day (mean)		36.4	42.3	1.009	0.998–1.020	NS
	3-month eGFR ml/min (mean)		71	56.7	0.977	0.963–0.990	0.001
	Number of 1st year readmissions (%)	< 3	87.3	73.9	1	–	–
		≥ 3	12.7	26.1	2.417	1.168–5.001	< 0.02
	Delayed graft function (%)	No	88.7	73.9	1	–	–
		Yes	11.3	26.1	2.755	1.322–5.739	0.007
	Acute kidney injury (%)	No	64.8	34.8	1	–	–
		Yes	35.2	65.2	3.455	1.814–6.578	< 0.001
	Acute rejection (%)	No	78.4	58.7	1	–	–
		Yes	21.6	41.3	2.553	1.349–4.833	< 0.005
	Infections (%)	No	25.8	13	1	–	–
		Yes	74.2	87	2.313	0.950–5.633	NS
	Urinary tract infection (%)	No	60.7	58.7	1	–	–
		Yes	39.3	41.3	1.085	0.582–1.025	NS
	CMV infection (%)	No	80.3	69.6	1	–	–
		Yes	19.7	30.4	1.787	0.906–3.526	NS
	Surgical complication (%)	No	82	80.4	1	–	–
		Yes	18	19.6	1.108	0.510–2.408	NS

*MMF* mycophenolate mofetil, *HLA* human leucocyte antigen, *MM* mismatch, *eGFR* estimated glomerular filtration rate, *CMV* cytomegalovirus, *NS* not significant, *KT* kidney transplantation.

Donor age ≥ 45, Azathioprine therapy, first-year readmissions ≥ 3, delayed graft function (DGF), acute kidney injury (AKI), and acute rejection (AR) have a negative impact. Mycophenolate mofetil (MMF) therapy has a positive impact on graft survival. The 3-month estimated glomerular filtration rate (eGFR) was also a predictor of 5-year graft survival.

We retained five predictors of 5-year graft survival after the multivariate analysis which are:

- donor age;
- MMF therapy;
- 3-month eGFR;
- DGF;
- number of hospital readmissions during the first year.

The primary reasons for readmissions during the first year, in our patients, were predominantly related to infectious complications and alteration of graft function.

### Machine learning predictive models

#### Feature selection

As mentioned previously, we tried seven feature selection algorithms to select the most important variables (recipient characteristics, donor characteristics, immunological data, KT procedure, immunosuppressive treatment, and post-transplant characteristics). MMF therapy and early AKI were selected as important variables affecting 5-year graft survival by all the feature selection methods. Four variables were selected by more than half of the methods. These variables are the length of the 1st hospitalization, 3-month-eGFR, tacrolimus therapy, and donors’ age.

#### Performance of the developed models

Five models resulted in AUCs over 80%. The highest AUC (89.7%) was obtained in the model developed with XGB with the features selected by the RF algorithm. This model was retained as the final model.

#### The best model

**Figure 4:**
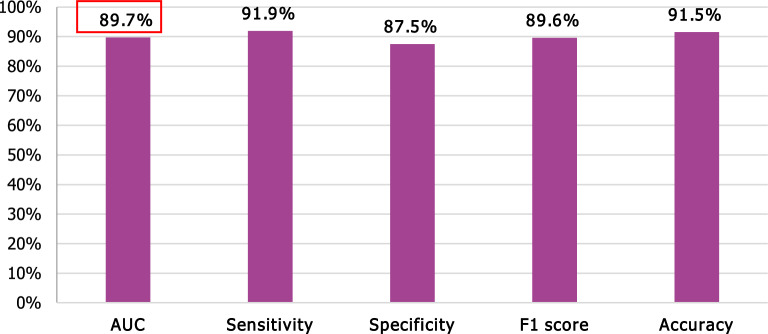
Performance measures of the best model.

Ten variables were selected as important variables affecting 5-year graft survival. Each feature was assigned an importance score that indicates how useful or valuable it was in the construction of the boosted decision trees within the model. The more an attribute is used to make key decisions with decision trees, the higher its relative importance is. The selected variables are in decreasing order of importance: Hypertension, history of red-blood-cell transfusion, early AKI post-KT, early AR, CMV infection, the length of the 1st hospitalization, MMF therapy, donor’s age, 3-month eGFR and the duration on dialysis before KT. The performance measures of the best model are summarized in Fig. 4.

## Discussion

An enhanced ability to predict graft survival at individual kidney transplant recipients is the key to improving KT outcomes, as it would help in providing more personalized clinical care. A predictive model of graft survival can be used as a decision tool for nephrologists to make therapeutic and counseling decisions for patients. Supposing that the proposed best model predicts a patient’s graft will fail within five years, a doctor may need to investigate ways to improve the chance of survival based on the patient profile (the selected predictors). Thus, safer and more personalized clinical practice guidelines can be developed.

The main limitation of our study is that data collection was retrospective which prevented us from considering some important variables because they weren’t available for all the patients, such as histological variables as we don’t practice protocol graft biopsy. Also, DSA screening wasn’t included as their detection in our center began in 2015, and our study period started in 1986. The limited number of patients is another weakness of our study.

The use of AI and the ML subtype in the world has been enabled by the explosion of numeric data available thanks to markedly enhanced computing power and cloud storage. With a sufficiently large database, an ML technique with appropriate algorithms can “learn” from the numerical correlations hidden in the dataset via a nonlinear fitting process and perform precise predictions. With such a technique, we don’t need to consider each variable separately, and we can directly acquire a precise prediction with a well-developed predictive model. ML is lying at the intersection of computer science and statistics.

While ML focuses on prediction by evaluating the conditional relations among variables, classical analyses often concentrate on survival estimation by identifying the most important predictors.

The most commonly used traditional statistical approaches in the field of organ transplantation are the Kaplan–Meier estimator, LR, and Cox proportional hazards models.

Classical statistical models, in general, assume the independence of the predictors. They are not designed to handle complex interactions among predictors and are not often used to model non-linear relationships among predictors and outcomes^11^. Added to the problem of statistical modeling assumptions such as linearity, normality, and equality of variance, these methods become ineffective when the number of predictors is large^8^.

Statistics make mathematical inferences about an output based on sample data. In statistics the question is: “is X related to Y?”. The goal of ML is to optimize predictive accuracy rather than inference. Hence, in ML the question is “given X, what is Y?”. In our study, we used both methods and each method resulted in a set of predictors (Table 2).

**Table 2:** Comparison between the results of machine learning and classical statistics.

Variable	Machine learning	Univariate LR*	Multivariate LR*
Donor age	**✓**	**✓**	**✓**
MMF therapy	**✓**	**✓**	**✓**
3-month eGFR	**✓**	**✓**	**✓**
Acute rejection	**✓**	**✓**	
Acute kidney injury	**✓**	**✓**	
CMV infection	**✓**		
Length of the 1st hospitalization	**✓**		
Hypertension	**✓**		
Transfusion	**✓**		
Dialysis duration	**✓**		
Readmissions 1st year		**✓**	**✓**
Delayed graft function		**✓**	**✓**
Azathioprine therapy		**✓**	
HLA MM A			
HLA MM DR			
Dialysis modality			
Proteinuria			

*MMF* mycophenolate mofetil, *eGFR* estimated glomerular filtration rate, *CMV* cytomegalovirus, *HLA* human leucocyte antigen, *MM* mismatch.

*Bivariate and multivariate logistic regression (SPSS 25).

Many authors used AI to predict KT outcomes. For short-term outcomes, Brier^12^ and Decruyenaere^13^ predicted DGF within the first week after KT. Shaikhina predicted acute antibody-mediated rejection at 30 days post-KT^14^.

For long-term graft survival (Table 3), the main endpoint over time in some of the published studies was defined as the time of graft failure by either returning a patient to dialysis or retransplantation^15^. Other studies developed models for a combined outcome of graft failure and death^11^. In our study, we only considered graft failure (death censored) for a more accurate prediction because graft survival and patient survival may have different predictors.

**Table 3:** Machine learning based studies for predicting 5-year kidney graft survival.

References	Target	Data size	Feature selection	Selected predictors	Model’s performance
Our study	5-year graft survival	407 KTs (living + deceased donors)	ML algorithm	History of hypertension, history of transfusion, duration on dialysis before KT, donor age, AKI post-KT, AR, CMV infection, length of the 1st hospitalization, 3-month eGFR, MMF therapy	AUC: 89.7% Se: 91% Sp: 87%
Loupy (2019), France^8^	Graft failure at different time points	4000 KTs (living + deceased donors)	Cox regression	Time of post-transplant risk evaluation, eGFR, proteinuria, histological parameters (interstitial fibrosis,tubular atrophy, glomerulitis, peritubular capillaritis, interstitial inflammation, tubulitis and transplant glomerulopathy) and DSA	Discrimination ability at 5 years (C index) 0.819 (95% confidence interval (0.799 to 0.839),
Nematollahi (2017), Iran^16^	Graft failure at 5 years post KT	717 KTs (living + deceased donors)	Clinical expertise and current available evidence	SCr at discharge, recipient age, donor age, donor blood group, cause of ESRD, recipient hypertension after KT and duration on dialysis before KT	Sn: 97.3% Sp: 26.1% Accuracy: 85.9% AUC: 76.9%
Shahmoradi (2016), Iran^17^	Graft survival at different time points	513 KTs (donor type not specified)	Not mentioned	Donor age, donor gender, recipient age, recipient gender, cause of ESRD, dialysis, duration on dialysis, panel test, BMI, donor type	Sn: 90.8% Sp: 52.0% Accuracy: 87.2%
Lofaro (2010), Italy^18^	Chronic allograft nephropathy at 5 years	80 KTs (living + deceased donors)	ML algorithm	Recipient age, number of transplants, 6-month eGFR, 6-month 24-h urine protein excretion, 6-month serum hemoglobin and 6-month hematocrit	Sn: 62.5% Sp: 92.8% AUC: 84.7%
Greco (2010), Italy^19^	Graft failure at 5 years	194 KTs (living + deceased donors)	Not mentioned	Recipient BMI, DGF, AR episode and chronic allograft nephropathy	Sn: 88.2% Sp: 73.8%
Akl (2008), Egypt^20^	Graft survival at 5 years	1900 KTs (living donors)	Univariate statistical analysis	Recipient age, donor age, transfusions, total HLA MM, HLA DR MM, Haplotype (sibling/related/unrelated donor), time to diuresis, total steroid dose (first 3 months), immunosuppression, acute tubular necrosis, AR episodes (first 3 months)	Sn: 88.4% Sp: 73.2% Accuracy: 95% AUC: 88%
Lin (2008), USA^11^	Graft survival at different time points including 5 years	57,383 KTs (living + deceased donors)	Clinical expertise and current available evidence	Recipient: age, gender, race, height, weight, cause of ESRD, history of hypertension, diabetes or CV disease, duration between date of current KT and failure date of the previous KT (if applicable), dialysis modality, predominant dialysis modality, and primary source of pay for treatment Donor: type, age, gender, race, height, weight and cause of death Number of matched HLA antigens, CIT and procedure type…	AUC: 77%
Krikov (2007), USA^21^	Graft survival at different time points including 5 years	92,844 KTs (living + deceased donors)	Survival analysis and multiple logistic legression	Recipient: race, gender, age, height, weight, multiple KT (yes/no), number of KTs, time on waiting list, predominant RRT modality, % on PD before KT, number of RRT modalities used before transplant, specific combination of RRT modalities, recipient comorbidity score, history of CV disease, history of unstable angina, history of diabetes, history of hypertension, VHB, VHC, peak and most recent level of panel reactive antibodies and primary source of pay for medical services Donor: race, gender, age, height, weight, donor type (living or deceased)	AUC: 71.7%

*KT* kidney transplantation, *AKI* acute kidney injury, *AR* acute rejection, *CMV* cytomegalovirus, *eGFR* estimated glomerular filtration rate, *MMF* mycophenolate mofetil, *DSA* donor specific antibody, *ESRD* end-stage renal disease, *BMI* body mass index, *HLA* human leucocyte antibody, *MM* mismatch, *CIT* cold ischemia time, *PD* peritoneal dialysis.

The main results of the published studies aiming to predict long-term graft survival are summarized in Table 3. Our model achieved a high AUC, ranking among the top performers in the reviewed studies. XGB, which is the training algorithm resulting in this model is a very powerful advanced ML algorithm. In order to confirm the reliability of our model we also performed cross-validation. These results are promising and the ML framework we used^10^ may give better results with larger databases.

The results of the existing studies are very encouraging, some of them are already validated and used as surrogate endpoint in clinical trials about long-term^22^. However, such big studies using advanced ML algorithms are awaiting in order to take advantages of AI superpower in this era of data abundance.

## Conclusions and perspectives

The use of ML in our study provided us with a reliable model enabling fast and precise prediction of 5-year graft survival using early, simple, non-invasive, and easily collectible variables with a good AUC (89.6%), high Se (91%), and a satisfying Sp (87%).

Our model relies on easily obtainable variables in the context of a developing country and does not necessitate invasive techniques such as graft biopsy.

Various features have been explored in the literature to predict 5-year graft survival (Table 3), categorized into pre-transplant and post-transplant attributes. Our database encompasses the most commonly encountered pre-transplant and post-transplant features, rendering our study comprehensive in its coverage of all facets of KT. Our model was trained using data from both living and deceased donors, with a focus on post-transplant features within the first year. This emphasis aligns with the fact that the most critical complications affecting long-term graft survival typically manifest during this initial period. Our approach aimed to construct a broadly applicable and comprehensive model capable of early prediction across various KT scenarios. While existing literature acknowledges statistical differences based on donor type, it does not compromise the model’s quality, as this factor may be considered as one of the influences on the outcome of interest: graft survival.

Regarding the donor’s features, the age of the donor appeared as an important factor that influences the prognosis of the long-term graft survival. All the studies that considered this factor have come to the same conclusion: the younger is the donor (whether alive or deceased), the better is graft survival^23–25^, which is ascertainable in our study population. DGF appeared also as a significant feature influencing long-term graft survival, despite a similar number of deceased donors between the groups. This disparity can be attributed to the influence of additional factors that contribute to DGF in KT from living donors, which appeared to have a significant impact on long-term survival. It is important to remind that, in our study, we defined DGF as the necessity for dialysis during the first week following KT.

In conclusion, our study leveraged the power of ML techniques to discern the most influential factors in long-term graft survival. Our model has enabled us to provide accurate predictions. However, it’s essential to recognize that our model’s reliability and generalizability must be substantiated through external validation. We are currently in the process of collaborating with the Tunisian Society of Nephrology, embarking on a multicentric study that encompasses all KT centers in Tunisia. This collaborative effort will significantly augment our dataset, enhancing the robustness and applicability of our predictive model.

## Data Availability

The datasets used and analyzed during the current study are available from the corresponding author on reasonable request.
